# Mechanical Principles Governing the Shapes of Dendritic Spines

**DOI:** 10.3389/fphys.2021.657074

**Published:** 2021-06-16

**Authors:** Haleh Alimohamadi, Miriam K. Bell, Shelley Halpain, Padmini Rangamani

**Affiliations:** ^1^Department of Mechanical and Aerospace Engineering, University of California, San Diego, La Jolla, CA, United States; ^2^Sanford Consortium for Regenerative Medicine, La Jolla, CA, United States; ^3^Section of Neurobiology, Division of Biological Sciences, University of California, San Diego, La Jolla, CA, United States

**Keywords:** lipid bilayer, dendritic spines, membrane-actin interactions, deviatoric curvature, tension

## Abstract

Dendritic spines are small, bulbous protrusions along the dendrites of neurons and are sites of excitatory postsynaptic activity. The morphology of spines has been implicated in their function in synaptic plasticity and their shapes have been well-characterized, but the potential mechanics underlying their shape development and maintenance have not yet been fully understood. In this work, we explore the mechanical principles that could underlie specific shapes using a minimal biophysical model of membrane-actin interactions. Using this model, we first identify the possible force regimes that give rise to the classic spine shapes—stubby, filopodia, thin, and mushroom-shaped spines. We also use this model to investigate how the spine neck might be stabilized using periodic rings of actin or associated proteins. Finally, we use this model to predict that the cooperation between force generation and ring structures can regulate the energy landscape of spine shapes across a wide range of tensions. Thus, our study provides insights into how mechanical aspects of actin-mediated force generation and tension can play critical roles in spine shape maintenance.

## 1. Introduction

Dendritic spines are small, bulbous protrusions along the dendrites of neurons that occur at postsynaptic glutamatergic synapses (Bosch and Hayashi, 2012; Nakahata and Yasuda, 2018; Nishiyama, 2019). They respond to a glutamate release by orchestrating a series of biochemical and biophysical events that span multiple spatial and temporal scales (Gray, 1959; Harris and Kater, 1994; Shepherd, 1996). Spine morphology is tightly coupled to synaptic function, with larger spines tending to represent stronger synapses (Arellano et al., 2007; Patterson and Yasuda, 2011) due to their greater surface expression of functional glutamate receptors. Synaptic activity regulates spine shape and volume. For example, several forms of physiological synaptic plasticity, such as long-term potentiation (LTP) and long-term depression (LTD) are associated with spine enlargement and spine shrinkage, respectively (Bear and Malenka, 1994; Engert and Bonhoeffer, 1999; Harris et al., 2003). Although average spine volume is approximately 0.1 femtoliter, the shape and volume of dendritic spines are highly variable, depending both on the developmental stage and a combination of genetic and environmental factors, including the prior history of activity (Fifkova, 1985; Harris, 1999; Ostroff et al., 2002; Petrak et al., 2005). Moreover, spine morphology is highly dynamic on the scale of seconds to minutes, due to a dynamic actin-based cytoskeleton (calabrese et al., 2006; Nakahata and Yasuda, 2018).

**Figure 1:**
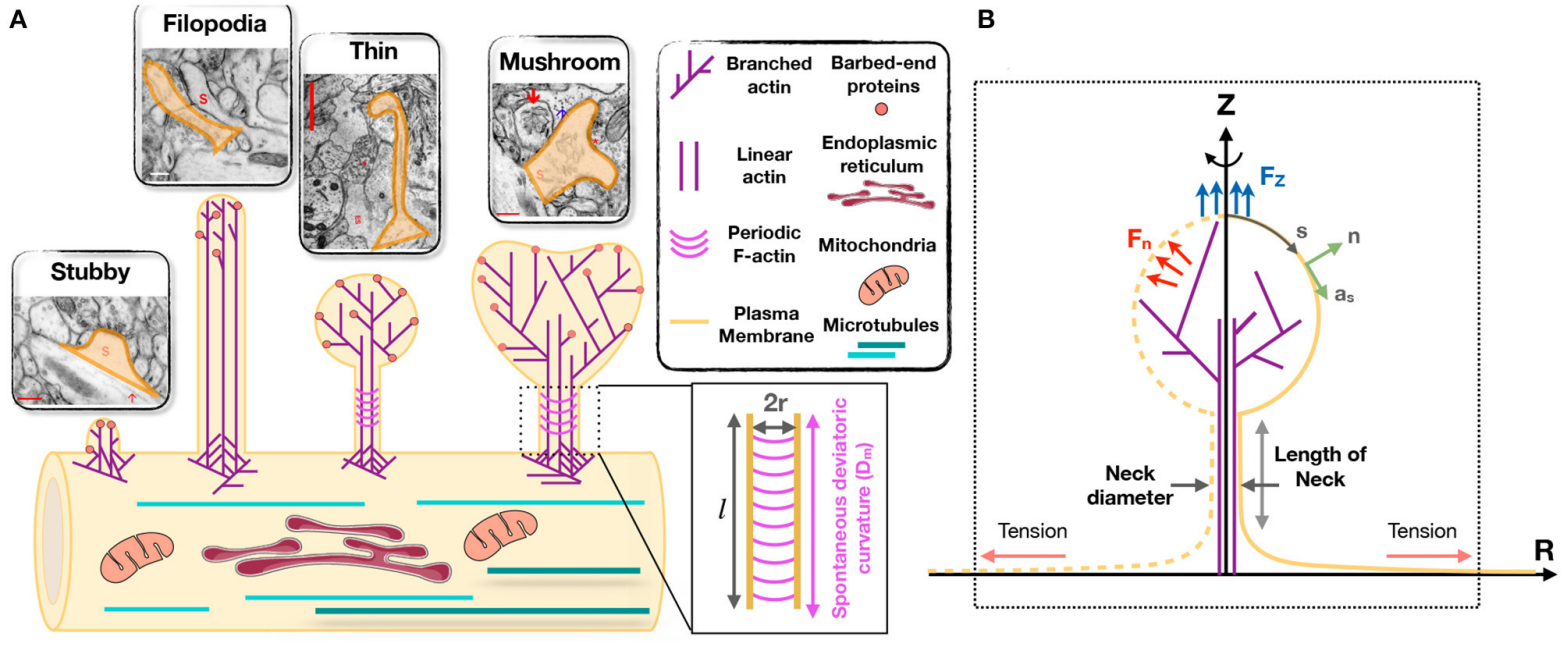
Modeling of forces relevant to spine shape. **(A)** Schematic depiction of different shape categories of dendritic spines (Reprinted with permission from SynapseWeb, Kristen M. Harris, PI, http://synapseweb.clm.utexas.edu/). The inset shows a schematic of a tubular neck with a radius r and a spontaneous deviatoric curvature D_m_ along the total neck length *l*. **(B)** The surface parametrization of the membrane geometry in axisymmetric coordinates. s is the arclength, **n** is the unit normal vector to the membrane surface, and **a**_*s*_ is the unit tangent vector in the direction of arclength. We assume that the actin filaments can apply axial (F_z_) or normal (F_n_) forces to the membrane surface. We assume that there is a large membrane reservoir with a fixed area, and we focused on the local region of the membrane under tension λ, as indicated by the dotted box.

Despite their broad range of morphological features and highly dynamic nature, dendritic spines can be classified into four broad categories. Spines in the mature nervous system are typically classified as being stubby, thin, or mushroom-shaped (Peters and Kaiserman-Abramof, 1970; Harris et al., 1992) (Figure 1A). These categories of spines can be identified in electron micrographs as postsynaptic structures connected to presynaptic nerve terminals. Stubby spines are short and wide, and lack a discernible neck. Such spines appear early during synaptogenesis and may represent an emerging spine, but they also might result from spine shrinkage driven by physiological or pathological conditions (Figure 1A) (Gray, 1959; Fiala et al., 1998; Harris, 1999).

The adult mammalian brain is dominated by either thin or mushroom-shaped spines. Thin spines have a long thin neck that is connected to a small bulbous head (Figure 1A) (Harris, 1999). Within the head is the postsynaptic density (PSD), an area just beneath the synaptic plasma membrane containing a high concentration of glutamate receptors, scaffolding molecules, and other proteins essential for postsynaptic function. Thin spines have flexible structures that allow them to adapt their morphology based on different levels of synaptic activity (Holtmaat et al., 2005; Zuo et al., 2005). It has been proposed that thin spines are “learning spines,” because they display a high capacity for expansion and strengthening via insertion of new AMPA-type glutamate receptors into the PSD, which is the key basis for synapse strengthening (Kasai et al., 2003; Holtmaat et al., 2005; Zuo et al., 2005; Bourne and Harris, 2007; Berry and Nedivi, 2017). Compared to thin spines, mushroom-shaped spines have a shorter neck and a greatly expanded head (Figure 1A) (Harris, 1999). Mature mushroom-shaped spines are more likely to be stable for months to years (Grutzendler et al., 2002; Trachtenberg et al., 2002; Holtmaat et al., 2005; Zuo et al., 2005; Berry and Nedivi, 2017), with slower turnover, and are associated with strong synapse functionality, as they contain on average higher concentrations of AMPA-type glutamate receptors. Such spines have therefore been called “memory spines,” in the sense that their potentiated strength reflects a history of high activity and thus “memory” storage, yet their capacity for further potentiation may be near saturation (Matsuzaki et al., 2001; Kasai et al., 2003; Ganeshina et al., 2004; Ashby et al., 2006; Bourne and Harris, 2007; Berry and Nedivi, 2017). Table 1 provides the reported dimensions for different shape categories of dendritic spines observed in hippocampal neurons (Harris et al., 1992; Spacek and Harris, 1997; Yuste and Bonhoeffer, 2004; Rodriguez et al., 2008; Kanjhan et al., 2016).

**Table 1:** Dimensions of different spine shapes compiled from the literature.

	**Stubby (Harris et al., 1992; Rodriguez et al., 2008)**	**Filopodia (Yuste and Bonhoeffer, 2004; Kanjhan et al., 2016)**	**Thin (Harris et al., 1992; Spacek and Harris, 1997; Rodriguez et al., 2008)**	**Mushroom (Harris et al., 1992; Spacek and Harris, 1997)**
Total length (L) (μm)	0.44 ± 0.15	2–20	0.98 ± 0.42	1.5 ± 0.25
Length of neck (*l*) (μm)	–	–	0.51 ± 0.34	0.43 ± 0.21
Neck diameter (2r) (μm)	0.32 ± 0.13	<0.3	0.1 ± 0.03	0.2 ± 0.07
Total volume (μm^3^)	0.03 ± 0.01	–	0.04 ± 0.02	0.29 ± 0.13
Volume of head (V) (μm^3^)	–	–	0.03 ± 0.15	0.27 ± 0.13
Total surface area (μm^2^)	0.45 ± 0.14	–	0.59 ± 0.29	2.7 ± 0.93
Surface area of head (μm^2^)	–	–	0.4 ± 0.15	2.4 ± 0.92
Surface area of PSD (μm^2^)	0.07 ± 0.02	–	0.05 ± 0.02	0.3 ± 0.1
Surface area of PSD/head	–	–	0.1 ± 0.06	0.18 ± 0.15

In addition to synapse-bearing spines, the fourth category of spine-like protrusions is dendritic filopodia. These are commonly observed during early development, and are thought to facilitate the pairing of presynaptic and postsynaptic glutamatergic sites during synaptogenesis by spatially scanning the neuropil volume for a partner axon (Miller and Peters, 1981; Dailey and Smith, 1996; Ziv and Smith, 1996; Fiala et al., 1998). Thus, a fraction of these “protospines” become synapse-bearing spines if they come into contact with and are stabilized in partnership with presynaptic nerve terminals (Dailey and Smith, 1996; Koleske, 2013). Filopodia are long (>2 μm) and thin (<0.3 μm diameter) protrusions that lack a bulbous head (Figure 1A) (Kanjhan et al., 2016).

Because the size and shape of functional subcellular domains are closely tied to the mechanics of actin-membrane interactions (Harris et al., 1992; Yuste and Bonhoeffer, 2004; Kanjhan et al., 2016), a more complete understanding of dendritic spine dynamics, development, and function would benefit from biophysical models that address the underlying mechanical aspects. We have therefore begun to build a computational model of spines that incorporates both membrane forces and actin-based forces, and their interaction. This model is based on published experimental observations in dendritic spines, non-neuronal cells, and biochemical experiments. The goal of this model is to inform our understanding of the development of spines and the plasticity of their structure under different physiological scenarios.

Currently, there are hundreds of studies that address various aspects of the regulation of dendritic spine size and shape. In building our model, we have chosen to focus on several key observations, as follows.

1. **Actin enrichment in spines:** Dendritic spines are enriched in filamentous actin, which, along with scaffolding molecules, establish spine architecture (Landis and Reese, 1983; Hotulainen and Hoogenraad, 2010; Bertling and Hotulainen, 2017). Membrane-actin interactions associated with spine enlargement and shrinkage during plasticity can be modeled at the single filament level using the elastic Brownian ratchet and the net force acting on the membrane due to actin remodeling can be represented as work done by actin to deform the membrane (Peskin et al., 1993; Mogilner and Oster, 1996; Miermans et al., 2017).
2. **Different subpopulations of actin:** There appear to be distinct subpopulations of F-actin in dendritic spines, and spine actin can be thought of as an independent network with interconnected nodes (Frost et al., 2010). The spine head typically consists of short, cross-linked filaments; branched filaments have been observed in the spine head (Hotulainen and Hoogenraad, 2010; Korobova and Svitkina, 2010; Nanguneri et al., 2019). The spine neck was initially thought to contain long filaments (Halpain, 2000; Rao and Craig, 2000; Tada and Sheng, 2006; Hotulainen et al., 2009), but current evidence has suggested the presence of short, branched filaments (Korobova and Svitkina, 2010). Additionally, recent high resolution imaging techniques have shown that there are likely periodic F-actin structures along the neck region of dendritic spines (Bär et al., 2016; Bucher et al., 2020). These periodic F-actin structures are very stable and in contrast to long and branched filaments, resist depolymerization (Bär et al., 2016).
3. **Membrane mechanics:** All cells regulate their shape by coordinating the properties of the cytoskeleton with that of the plasma membrane. Proteins such as MARCKS that interact directly with both F-actin and the lipid bilayer can strongly influence spine shape (Calabrese and Halpain, 2005). Membrane curvature is especially important in spines and represents a specific mechanical force that is regulated by distribution of proteins and lipids. Additionally, membrane composition can regulate transport phenomena between two adjacent cells (Rahmaninejad and Vaughan, 2021). Bin/Amphiphysin/Rvs (BAR)-domain containing proteins assemble on the membrane to produce anisotropic curvature and promote tubulation. Studies have demonstrated critical roles for specific BAR-domain proteins in dendritic spines. Recently, the role of membrane mechanics has been elucidated in the initiation of dendritic spines (Hlushchenko et al., 2016). A series of studies showed that dendritic spines can be initiated by membrane bending due to protein patches containing BAR domains such as I-BAR and F-BAR proteins (Carlson et al., 2011; Wakita et al., 2011; Kessels and Qualmann, 2015; Saarikangas et al., 2015). These proteins are known to polymerize on the membrane (Peter et al., 2004; Shimada et al., 2007; Frost et al., 2008, 2009), induce anisotropic curvature (Kralj-Iglič et al., 2000; Iglič et al., 2005, 2007), and promote tubulation (Kralj-Iglič et al., 1996, 1999; Iglič et al., 1999; Frost et al., 2008; Kabaso et al., 2012).

The above findings suggest that membrane bending and actin-membrane interactions are major determinants of spine morphology. Recent studies have modeled the role of either membrane mechanics alone (Miermans et al., 2017) or actin dynamics alone in spines (Bonilla-Quintana et al., 2020), but the interaction between the two has not yet been addressed. Here, we present a general theoretical model that relates membrane bending and actin-mediated forces to spine morphology. Using this model, we investigate the mechanical landscape of the different shapes of spines and map the relationships among actin-mediated force generation, membrane elasticity, and curvature induced by periodic ring structures and proteins such as BAR domains.

## 2. Model Development

### 2.1. Assumptions

- We treat the lipid bilayer as a continuous thin elastic shell, assuming that the membrane thickness is negligible compared to the radii of membrane curvature (Helfrich, 1973; Deuling and Helfrich, 1976). This allows us to model the bending energy of the membrane using the modified version of the Helfrich-Canham energy, including the effect of spatially varying deviatoric curvature to represent the induced anisotropic curvatures by periodic F-actin rings and other ring-shaped structures (Canham, 1970; Helfrich, 1973; Kralj-Iglič et al., 1996, 1999; Iglič et al., 2006; Alimohamadi and Rangamani, 2018).
- We assume that the membrane is locally inextensible, since the stretching energy of the lipid bilayer is an order of magnitude larger than the membrane bending energy (Rawicz et al., 2000). We implemented this constraint using a Lagrange multiplier, which can be interpreted as the membrane tension (Steigmann, 1999; Alimohamadi et al., 2020a). We note that this membrane tension, in this study, is better interpreted as the cortical tension including the effective contribution of the membrane in-plane stresses, induced tension by actin polymerization, and myosin-driven contractility against membrane (Barfod et al., 2011; Diz-Muñoz et al., 2013; Orly et al., 2014; Alimohamadi et al., 2020b).
- We assume that the time scales of mechanical forces are much faster than other events (such as actin polymerization) in dendritic spines, allowing us to assume mechanical equilibrium and neglect inertia (Steigmann, 1999; Miermans et al., 2017). This assumption is justified by the fact that the timescale of the equilibration of the mechanical forces is much smaller than the timescale of actin polymerization in dendritic spines (Weichsel and Geissler, 2016).
- We assume that the force exerted by the actin cytoskeleton can be represented as work done on the membrane and do not include the molecular details of the actin network (Atilgan et al., 2006; Walani et al., 2015; Miermans et al., 2017; Alimohamadi et al., 2020b). Additionally, we assume that the periodic ring shaped structures of actin and related proteins such as βII spectrin and BAR-domain proteins can be represented using an anisotropic spontaneous curvature (Kralj-Iglič et al., 1996, 1999; Iglič et al., 2006, 2007).
- For ease of computation we assume that the geometry of a dendritic spine is rotationally symmetric (see Figure 1B) (Miermans et al., 2017). This assumption allows us to parametrize the whole surface by a single parameter, arclength.

### 2.2. Mechanical Force Balance

In this section, we present a concise derivation of the governing mathematical shape equations for the shape of dendritic spines at mechanical equilibrium. The complete derivation with details is given in Steigmann (1999), Agrawal and Steigmann (2009), and Walani et al. (2015). The total free energy of the system (*E*) includes the elastic storage energy of the membrane (*E*_elastic_), and the work done by the applied forces due to actin filaments (*W*_force_) (Lokar et al., 2012; Walani et al., 2014, 2015; Alimohamadi and Rangamani, 2018) is given by

(1) $$\begin{array}{l} E=E_{\text{elastic}}-W_{\text{force}}, \end{array}$$

(2a) $$\begin{array}{l} E_{\text{elastic}}=\int_{\omega }\left(\sigma \left(H,D;\theta^{\alpha }\right)+\lambda \left(\theta^{\alpha }\right)\right)da-pV,\text{ and} \end{array}$$

(2b) $$\begin{array}{l} W_{\text{force}}=\int_{\omega }\text{f}\left(\theta^{\alpha }\right)\cdot \left(\text{r}-{\text{r}}_{0}\right)da. \end{array}$$

Here, ω is the total membrane surface area, σ is the bending energy density per unit area, θ^α^ denotes the surface coordinate where α ∈ {1, 2}, *H* is the mean curvature of the surface, *D* is the curvature deviator, *K* is the Gaussian curvature of the surface, λ is the tension field and represents the Lagrange multiplier associated with the local area constraint, *p* is the transmembrane pressure and represents the Lagrange multiplier associated with the volume constraint, *V* is the enclosed volume, **f** is the applied force per unit area, **r** is the position vector in the current configuration, and **r**_0_ is the position vector in the reference frame. To model the energy density σ in Equation (2a), we used the modified version of Helfrich energy including the effects of induced anisotropic curvature by periodic F-actin structures and BAR domain proteins (Canham, 1970; Helfrich, 1973; Iglič et al., 2005; Alimohamadi and Rangamani, 2018; Alimohamadi et al., 2018), given as

(3) $$\begin{array}{l} \sigma \left(H,D;\theta^{\alpha }\right)=\left(2k_{1}+k_{2}\right)H^{2}-k_{2}{\left(D-{\text{D}}_{\text{m}}\left(\theta^{\alpha }\right)\right)}^{2}, \end{array}$$

where *k*_1_ and *k*_2_ are constants and D_m_ is the spontaneous (intrinsic) deviatoric curvature which can be spatially heterogeneous along the membrane surface (Kralj-Iglič et al., 1996, 1999; Iglič et al., 2005). For an isotropic case (D_m_ = 0), Equation (3) reduces to the classical Helfrich energy with quadratic dependence on mean curvature and linear dependence on Gaussian curvature (Iglič et al., 2005), where *k*_1_ = κ (bending modulus) and *k*_2_ = κ_*G*_ (Gaussian modulus). In this study, we assume κ_*G*_ ~ −κ (Hu et al., 2012) and simplify the bending energy density in Equation (3) as (Iglič et al., 2005; Walani et al., 2014)

(4) $$\begin{array}{l} \sigma \left(H,D;\theta^{\alpha }\right)=\kappa H^{2}+\kappa {\left(D-{\text{D}}_{\text{m}}\left(\theta^{\alpha }\right)\right)}^{2}. \end{array}$$

It should be mentioned that in Equation (4), we assumed that periodic rings can only induce anisotropic curvature and we set the isotropic curvature (spontaneous curvature) to be zero throughout this study. Substituting Equations (2a), (2b), and (4) into Equation (1) gives

(5) $$\begin{array}{l} E=\underset{\begin{array}{l} \text{Bending energy }\\ \text{of the membrane} \end{array}}{\underset{︸}{\int_{\omega }\kappa H^{2}da}}+\underset{\begin{array}{l} \text{Bending energy due to }\\ \text{deviatoric curvature} \end{array}}{\underset{︸}{\int_{\omega }\kappa {\left(D-{\text{D}}_{\text{m}}\right)}^{2}da}}\;+\underset{\begin{array}{l} \text{Work done}\\ \text{by tension} \end{array}}{\underset{︸}{\int_{\omega }\lambda da}}\text{- }\underset{\begin{array}{l} \text{Work done}\\ \text{by pressure} \end{array}}{\underset{︸}{\text{pV}}}\\ \;-\underset{\text{Work done by actin-mediated forces}}{\underset{︸}{\int_{\omega }f(\theta^{\alpha })\cdot (r-r_{0})da}} \end{array}$$

Minimization of the energy (Equation 5) using the variational approach results in the governing shape equation (Supplementary Equation 6) and the incompressibility condition (Supplementary Equation 7) for a heterogeneous membrane. The complete equations are presented in the Supplementary Material along with the complete notation in Supplementary Table 1.

### 2.3. Numerical Implementation

In axisymmetric coordinates, the membrane shape equation (Supplementary Equation 6) and the incompressibility condition (Supplementary Equation 7) simplify to a coupled system of first order differential equations (Supplementary Equation 20). In order to solve this system of equations along with the prescribed boundary conditions (Supplementary Equation 22), we used “bvp4c,” a boundary value problem solver in MATLAB. In all our simulations, we assume that the total area of the membrane is conserved and we also fixed the bending modulus to be κ = 0.18 pN·μm based on previous models for spines (Pontes et al., 2013; Bonilla-Quintana et al., 2020). We also set the transmembrane pressure to zero (*p* = 0) to focus only on the mechanism of membrane-actin interactions in governing the shapes of dendritic spines.

## 3. Results

Using the model described above, we conducted simulations for different mechanical parameters with the goal of identifying the range of forces, the associated heterogeneities, and the protein-induced and cytoskeleton-induced anisotropic curvatures that could result in shapes and sizes of spines corresponding to those observed experimentally (Table 1). Specifically, we sought to recreate the filopodial, stubby, thin, and mushroom-shaped spines as shown in Figure 1. We must emphasize that all the shapes are equilibrium shapes, and our model does not provide insight into dynamic transitions from one shape to another. Our simulation results are described below. In these data, we emphasize the relationships among different mechanical parameters to obtain the desired shapes, and give specific values for mechanical parameters that result in sizes as listed in Table 1. These provide some realistic magnitudes for forces present at various locations within the compact spine volume.

### 3.1. Localized Axial Forces Along the Membrane Are Sufficient for the Formation of Stubby and Filopodial Shaped Spines

**Figure 2:**
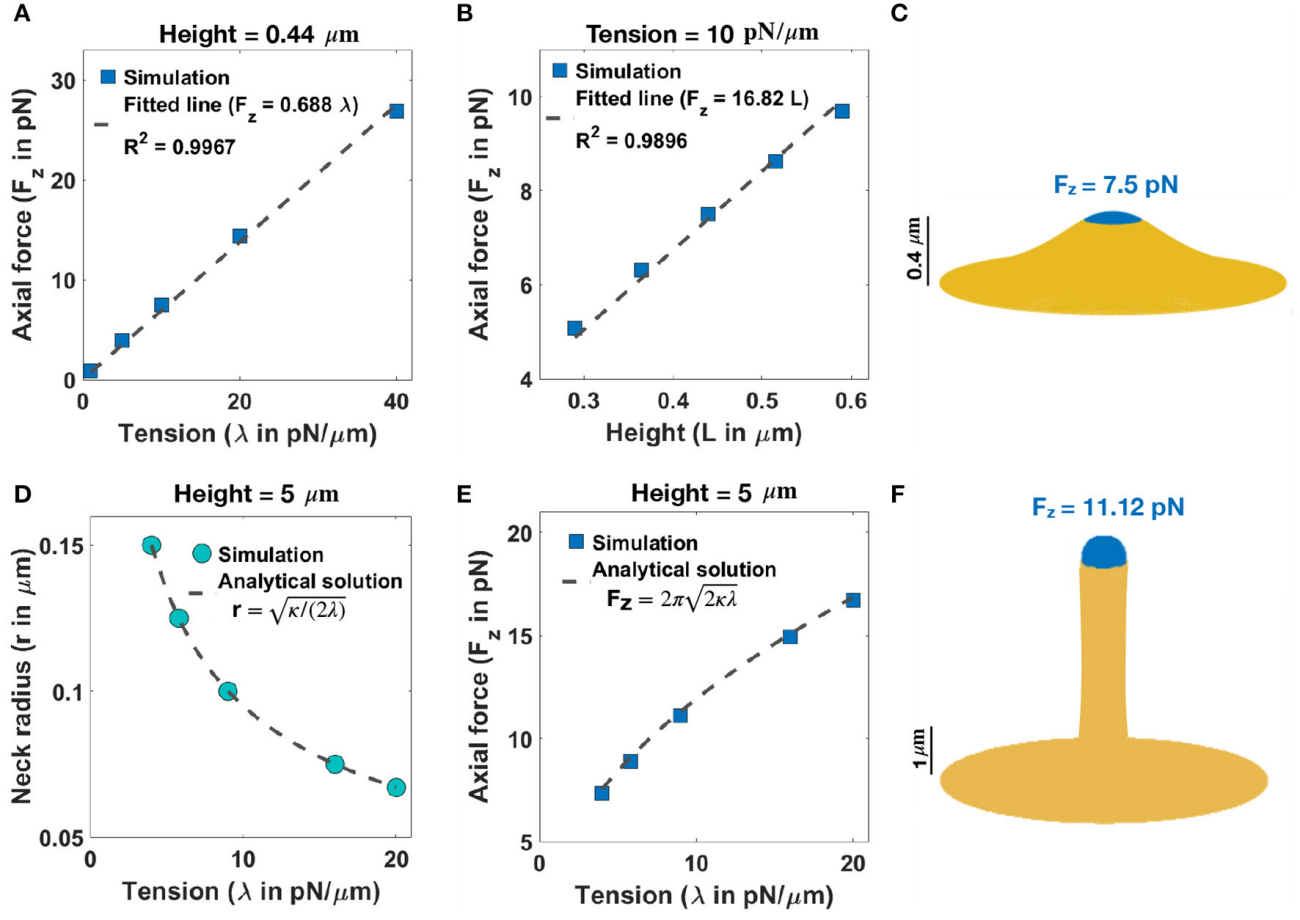
Formation of stubby and filopodia shaped spines with a localized axial force. **(A)** Linear relationship between the magnitude of axial force and tension in a small stubby-shaped membrane deformation (Derényi et al., 2002; Powers et al., 2002). The dashed line is the fitted curve (F_z_ = 0.688λ) with R^2^ = 0.9967. **(B)** Linear relationship between the magnitude of axial force and the height of the stubby spine for a fixed tension (Derényi et al., 2002; Powers et al., 2002). The dashed line is the fitted curve (F_z_ = 16.82L) with R^2^ = 0.9896. **(C)** A stubby-shaped spine with a total length *L* = 0.44 μm is formed with F_*z*_ = 7.5 pN applied along the blue area (λ = 10 pN/μm). **(D)** Neck radius of a filopodium as a function of tension (*r* $=\sqrt{\kappa /\left(2\lambda \right)}$) (Derényi et al., 2002). **(E)** The magnitude of axial force needed to form a filopodium as a function of tension (${\text{F}}_{\text{z}}=2\pi \sqrt{2\kappa \lambda }$) (Derényi et al., 2002). **(F)** A filopodium-shaped protrusion with a total length *L* = 5 μm and neck radius *r* = 0.2 μm is formed with F_*z*_ = 11.2 pN applied along the spherical cap of the filopodium, which is shown in blue (λ = 9 pN/μm).

We begin with an analysis of the force-shape relationship of stubby spines. We assumed that actin filaments exert axial forces in the nascent PSD area, which is a small fraction of the membrane surface area (Table 1). This heterogeneous force distribution along the membrane was implemented using a hyperbolic tangent function (Supplementary Equation 23). We observed that the relationship between the magnitude of the forces and the length of the stubby spines depends on the value of tension. To map this relationship, we performed the simulation for (*i*) a fixed height (*L* = 0.44 μm) and a wide range of tensions (Figure 2A) and (*ii*) a fixed tension (e.g., λ = 10 pN/μm) and different heights of the stubby spine (Figure 2B). As shown in previous studies (Derényi et al., 2002; Powers et al., 2002), for a small membrane deformation, such as a stubby spine, the axial force is linearly proportional to both tension and the height of the stubby spine (Figures 2A,B) (Derényi et al., 2002; Powers et al., 2002). Thus, from a mechanical standpoint, the stubby spine shape is accessible for a wide range of forces and tensions in the physiological range. For example, based on our simulation, when tension is λ = 10 pN/μm, an axial force of F_z_ = 7.5 pN is required to form a stubby spine of the length of *L* = 0.44 μm (Table 1 and Figure 2C).

Next, we investigated the role of forces in the formation of long spines that resembled filopodia. For the simplest case with no steric interaction between membrane and bundled actin, we found that the formation of a long filopodium follows well-established results for tube formation from a membrane reservoir (Derényi et al., 2002). Ignoring the spherical cap, a filopodium is a tubular membrane and its equilibrium radius (*r*) depends on the tension and bending rigidity of the membrane as *r* $=\sqrt{\kappa /\left(2\lambda \right)}$ (Figure 2D) (Derényi et al., 2002). The axial force F_z_ required to maintain the tubule with radius *r* is given as ${\text{F}}_{\text{z}}=2\pi \sqrt{2\kappa \lambda }$ (Figures 2E,F) (Derényi et al., 2002), which is independent of the length of the protrusion (Supplementary Figure 3A). In addition to the actin-mediated filopodium formation from a large membrane reservoir (fixed membrane area) that we focused on here, Miermans et al. showed that an increase in the surface area of a spine can drive a filopodium elongation from a stubby-shaped spine (Miermans et al., 2017). They suggested that exocytosis of endosomes at synapses provides this membrane addition to the system (Miermans et al., 2017).

### 3.2. Normal Forces Along the Membrane Support the Formation of Thin Shaped Spines

We next investigated the nature of forces that could be associated with the formation of thin-shaped spines. Because thin-shaped spines have a bulbous head, axial forces such as those used in Figure 2 are insufficient to generate the spherical shape of the head. Since spherical shapes can be obtained by a normal force acting locally on the head region, we repeated the simulation in Figure 2 but now included a localized uniform normal force density along the area of the spine head (*A*_force_ = *A*_spine head_). It is possible that such normal forces result from the dense actin meshwork in the spine heads (Borisy and Svitkina, 2000; Miermans et al., 2017). We estimated the forces required to generate a spherical head by assuming that a thin spine is ideally a sphere with radius R which is connected to a cylinder with radius *r* and height *l* (Supplementary Figure 1B). If a uniform normal force density, f_n_, is applied all along the sphere, then, ignoring the interface between the sphere and the cylinder, the total energy of the system can be written as

(6) $$\begin{array}{l} E=E_{\text{sphere}}+E_{\text{cylinder}}, \end{array}$$

where *E*_sphere_ =$\;\left(\kappa /R^{2}+\lambda \right)4\pi R^{2}-\left(4\pi /3\right)R^{3}{\text{f}}_{\text{n}}$ and *E*_cylinder_ =$\;2\pi \sqrt{2\lambda \kappa }l$ (see section 1.5.2 in the Supplementary Material). Minimizing the total energy of the system with respect to R by taking ∂*E*/∂*R* = 0, we obtain the equilibrium normal force density as f_n_ = 2λ/*R*. This resembles the Young-Laplace equation where normally pressure (normal force density) is a global parameter; in this case, f_n_ is a local normal force density. In our simulation, we prescribe the area of the applied force and thus we can rewrite the force density as

(7) $$\begin{array}{l} {\text{f}}_{\text{n}}=4\lambda \sqrt{\frac{\pi }{{\text{A}}_{\text{force}}}}. \end{array}$$

**Figure 3:**
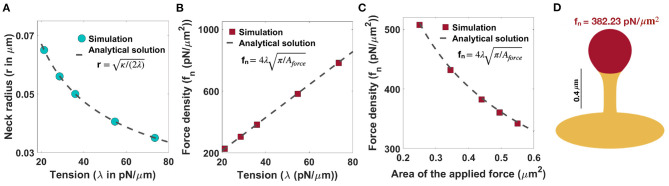
Formation of thin-shaped spines with localized normal force density along the spine head. **(A)** Neck radius of a thin-shaped spine as a function of tension (*r* $=\sqrt{\kappa /\left(2\lambda \right)}$) (Derényi et al., 2002). **(B)** Linear relationship between the magnitude of normal force density needed to form a thin-shaped spine and the tension. Here, the area of the applied force is set at *A*_force_ = 0.44 μm^2^. The red squares represent the results obtained from simulation and the dashed line is the derived analytical solution (${\text{f}}_{\text{n}}=4\lambda \sqrt{\pi /A_{\text{force}}}$, Equation (7)). **(C)** The magnitude of a normal force density needed to form a thin-shaped spine as a function of the area of the spine head. The tension is set at λ = 36 pN/μm. The red squares represent the results obtained from our simulations and the dashed line is the derived analytical solution ($({\text{f}}_{\text{n}}=4\lambda \sqrt{\pi /A_{\text{force}}}$, Equation (7). **(D)** A thin-shaped spine with a total length *L* = 0.98 μm, neck radius *r* = 0.05 μm, and head volume *V* = 0.033 μm^3^ is formed with f_n_ = 382.23 pN/μm^2^ applied along the head of spine which is shown in red (λ = 36 pN/μm and *A*_force_ = 0.44 μm^2^).

In order to generate thin-shaped spines, we first fixed the neck diameter based on the magnitude of tension (*r* $=\sqrt{\kappa /\left(2\lambda \right)}$) as shown in Figure 2D. Similar to filopodia, in thin spines, the radius of the neck is related to the tension and the bending rigidity, given by *r* $=\sqrt{\kappa /\left(2\lambda \right)}$ (Derényi et al., 2002) (Figure 3A). This relationship suggests that in order to have a thin spine with a neck radius between 0.035 μm < *r* <0.065 μm (given range in Table 1), the tension can vary between 20 pN/μm < λ < 80 pN/μm. Based on Equation (7), the magnitude of the normal force density linearly depends on the tension, while it varies as the inverse of the square root of the area of applied force.

In Figure 3B, we plotted the magnitude of the normal force density as a function of tension obtained from numerical solutions (red squares) vs. the analytical expression given in Equation (7) (dotted line) for fixed *A*_force_ = 0.44 μm^2^. We found a good agreement between the analytical solution and the results obtained from simulation such that by changing tension between 20pN/μm < λ < 80pN/μm, the magnitude of the normal force density required to form a thin-shaped spine varies in a large range between $200\text{pN}/\text{μ}{\text{m}}^{2}<{\text{f}}_{\text{n}}<900\text{pN}/\text{μ}{\text{m}}^{2}$ (Figure 3B). To further validate our numerical results, we plotted the magnitude of the normal force density as a function of the area of the applied force (*A*_force_) obtained from numerical solution (red squares) vs. the analytical expression given in Equation (7) (dotted line) for a fixed tension, λ = 36 pN/μm (Figure 3C). We observed a good agreement between the analytical solution and the numerical results where by increasing the area of the applied force from $A_{\text{force}}=0.25\text{μ}{\text{m}}^{2}$ to $A_{\text{force}}=0.55\text{μ}{\text{m}}^{2}$, the magnitude of the normal applied force density needed to form a thin spine decreases from f_n_ ~ 500 pN/μm^2^ to f_n_ ~ 300 pN/μm^2^ (Figure 3C).

As an example, to form a thin spine with an average neck diameter of *r* = 0.05 μm (see Table 1), we set our tension to be λ = 36 pN/μm (r $=\sqrt{\kappa /\left(2\lambda \right)}$). Based on our calculation for λ = 36 pN/μm and *A*_force_ = 0.44 μm^2^ (average area of the spine head in Table 1), a total normal force density of f_n_ = 382.23 pN/μm^2^ (applied along the red area) is required to form a thin spine with a total length *L* = 0.98 μm, a neck radius *r* = 0.05 μm, and a head volume *V* = 0.033 μm^3^ (Figure 3D). Also, in Supplementary Figure 3B, we show that the magnitude of the normal force density needed to form a thin spine is independent of the height of the spine.

### 3.3. Non-uniform Normal Force Distributions Can Result in Mushroom-Shaped Spines

**Figure 4:**
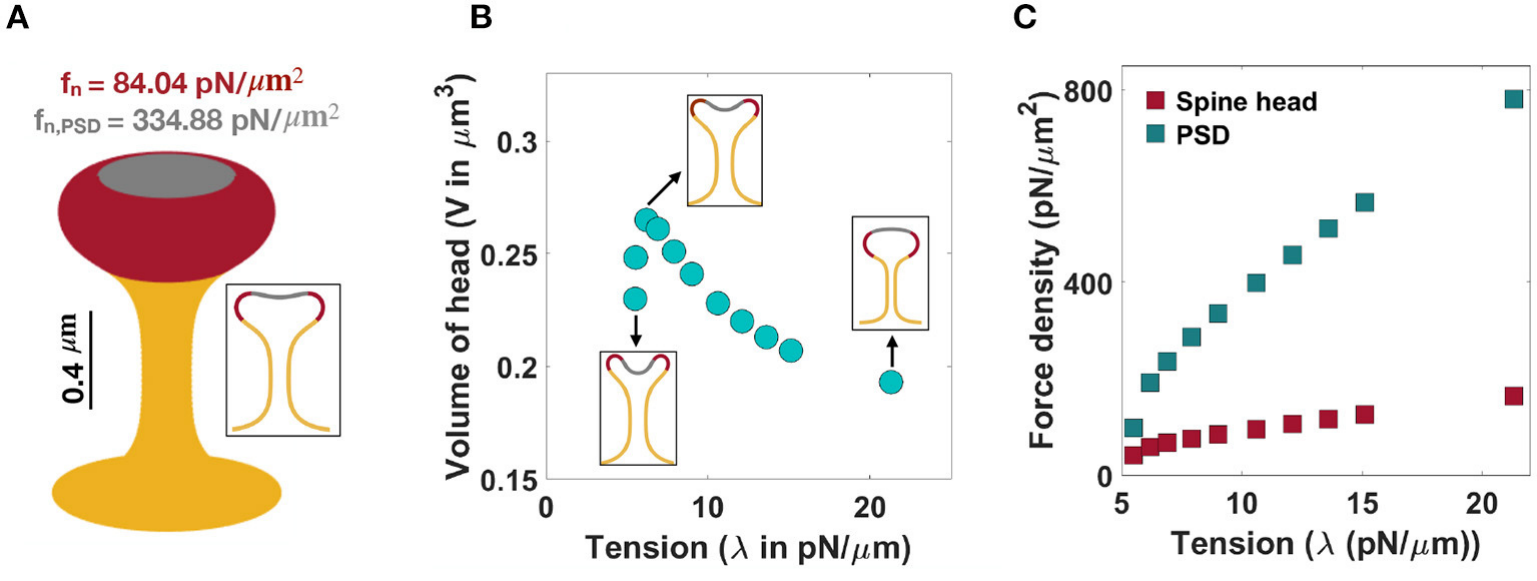
Formation of mushroom-shaped spines with localized normal forces along the spine head and PSD. **(A)** A mushroom-shaped spine with a total length *L* = 1.51 μm, neck radius *r* = 0.1 μm, head volume *V* = 0.25 μm^3^, and area of PSD/area of head = 0.2 is formed with f_n_ = 84.04 pN/μm^2^ applied along the head of spine (red domain) and f_n,PSD_ = 334.88 pN/μm^2^ applied along the PSD (gray domain) (λ = 9 pN/μm). **(B)** The non-monotonic behavior of the volume of a mushroom-shaped spine head when increasing tension. Three different shapes of mushroom-shaped spines are shown for low, intermediate, and high tensions. With increasing magnitude of tension, the mushroom-shaped spine head flattens. **(C)** The magnitude of normal force densities in the spine head (red squares) and in PSD (gray squares) increases with increasing tension.

We next asked if changes to the force distributions could result in mushroom-shaped spines. We hypothesized that one possible way is to have a heterogeneous force distribution along the spine head and the PSD area. The heterogeneous force distribution assumption comes in part from the observation that the presence of the presynaptic terminal might cause the concave structure of the PSD on the dendritic spine, suggesting the possibility that the net force on the membrane would be altered at the PSD region (Kashiwagi et al., 2019). To understand how non-uniform distributions of normal forces can characterize the morphology of mushroom spines, we performed simulations assuming that the normal force applied along the PSD area is different from the normal force density applied along the rest of the spine head (Figure 4A).

In the case of mushroom-shaped spines, we have multiple geometric parameters to consider—(a) head volume, (b) area fraction of the PSD, and (c) neck diameter determined by tension. For example, to form a mushroom-shaped spine with a total length *L* = 1.51 μm, head volume *V* = 0.25 μm^3^, and area of PSD/ area of head ratio = 0.2 (see Table 1), normal force densities of f_n_ = 84.04 pN/μm^2^ and f_n,PSD_ = 334.88 pN/μm^2^ are required along the spine head (red region) and the PSD area (gray region), respectively (Figure 4A). The value of tension was set to λ = 9 pN/μm to obtain a neck radius of about r ≈ 0.1 μm (see Table 1 and Supplementary Figure 4). The magnitude of these force densities is independent of the height of the spine (Supplementary Figure 3C).

We observed that the morphology of the spine head changes with varying magnitude of tension; the spine head flattens for large tensions (Figure 4B). This is consistent with previous studies that have investigated membrane shape at high tensions, e.g., the membrane remains almost flat during vesicle budding (Saleem et al., 2015), or in the case of a red blood cell, the biconcave cell flattens to a pancake shape (Evans, 2018; Alimohamadi et al., 2020b). To further investigate how a change in the morphology of the spine head can affect the volume of the head, we plotted the volume of the head (V) as a function of tension (Figure 4B). We found that the head volume is a non-monotonic function of tension; as tension increases, the volume of the spine head increases and then decreases (Figure 4B).

This is because initially when increasing tension from low to intermediate values the head flattens and the volume of the head increases. However, for high tensions, the shrinkage of the head becomes dominant and as a result the volume decreases (Figure 4B). Consistent with these observations, a larger normal force is required to bend a stiffer membrane and form a mushroom-shaped spine (Figure 4C). For example, based on our calculation, when increasing tension from λ = 5 pN/μm to λ = 20 pN/μm, the normal force densities in the spine head and PSD area increase by almost 120 and 680 pN/μm^2^, respectively (Figure 4C).

To study how the ratio of PSD area to the total area of the spine head affects the magnitude of normal force densities, we performed simulations for a range of area of PSD/area of head ratios (Supplementary Figure 5). Our results show that with increasing area of PSD/area of head ratio, a larger normal force density in the spine head and a smaller normal force in the PSD region are required (Supplementary Figure 5A). Additionally, increasing the ratio of the PSD area to the total area of the head results in the flattening of the spine head with a larger volume (Supplementary Figure 5B). Thus, mushroom-shaped spines can be formed from a multitude of mechanical pathways—heterogeneous forces in the spine head, balancing tension and force distributions, and using different area localizations of the forces.

### 3.4. Induced Spontaneous Deviatoric Curvature by Periodic F-Actins Structures and BAR Domain Proteins Can Generate Characteristic Dendritic Spine Necks

Recently, super-resolution microscopy methods have revealed the presence of ubiquitous actin ring structures along spine necks (Bär et al., 2016; Bucher et al., 2020). It has been suggested that these ring-like structures and BAR-domain proteins can together support the tubular shape of dendritic spines (Ebrahimi and Okabe, 2014; Bertling and Hotulainen, 2017). To understand how periodic F-actin structures and BAR domain proteins can regulate the tubular shape of spine necks, we implemented their net effect in our model by including spontaneous deviatoric curvature in the energy density of the system (Equation 4) (Kralj-Iglič et al., 1996, 1999; Iglič et al., 1999, 2005; Kabaso et al., 2012).

**Figure 5:**
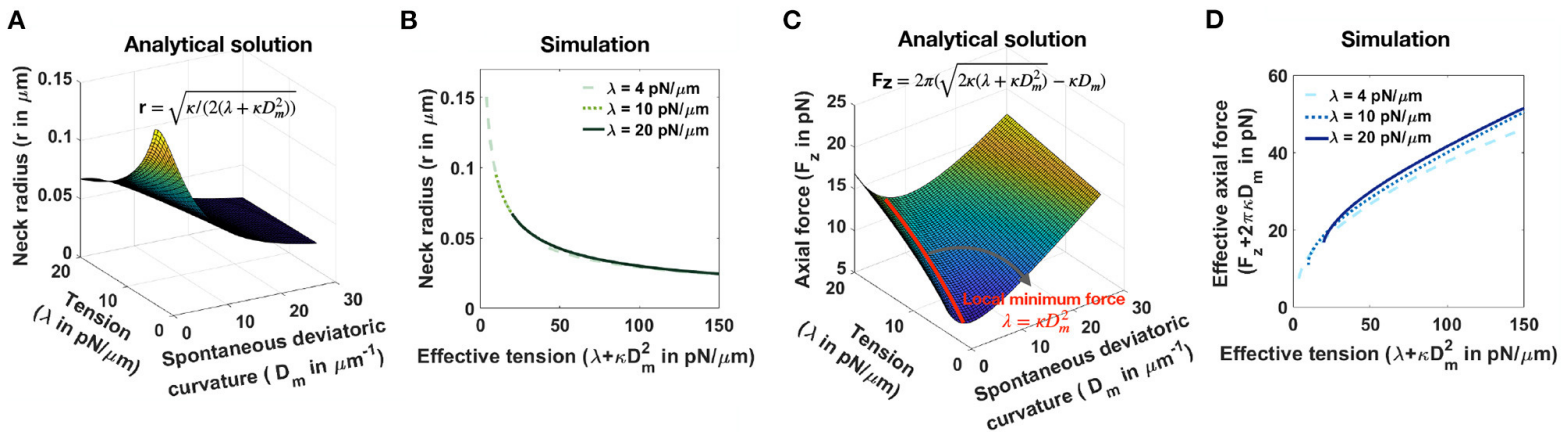
Effective tension including spontaneous deviatoric curvature regulates the neck radius and the magnitude of axial force in a tubular membrane. **(A)** Analytical solution for the neck radius of a tubular membrane as a function of spontaneous deviatoric curvature and tension ($\text{r}=\sqrt{\kappa /\left(2\left(\lambda +\kappa {\text{D}}_{\text{m}}^{2}\right)\right)}$, Supplementary Equation 37). **(B)** The neck radius obtained from numerical solutions as a function of effective tension ($\lambda +\kappa {\text{D}}_{\text{m}}^{2}$). Here, for fixed three different tensions, we varied the effective tension by changing the spontaneous deviatoric curvature between $0<{\text{D}}_{\text{m}}<30\text{μ}{\text{m}}^{-1}$. The radii of the membrane necks collapse onto a single curve for different tensions. **(C)** Analytical solution for the magnitude of an axial force needed to maintain a tubular protrusion as a function of spontaneous deviatoric curvature and tension (${\text{F}}_{\text{z}}=2\pi \left(\sqrt{2\kappa \left(\lambda +\kappa {\text{D}}_{\text{m}}^{2}\right)}-\kappa {\text{D}}_{\text{m}}\right)$, Supplementary Equation 37). The axial force needed to maintain a tubular protrusion has a local minimum along the red line where λ =$\;\kappa {\text{D}}_{\text{m}}^{2}$ (Supplementary Equation 39). **(D)** The effective axial force (F_z_+2πκD_m_) obtained from numerical solutions as a function of effective tension ($\lambda +\kappa {\text{D}}_{\text{m}}^{2}$). Here, for fixed three different tensions, we varied the effective tension by changing the spontaneous deviatoric curvature between $0<{\text{D}}_{\text{m}}<30\text{μ}{\text{m}}^{-1}$. Effective axial forces collapse onto a single curve for different tensions.

Consider a tubular membrane with radius *r* and a spontaneous deviatoric curvature D_m_ along the neck with total length *l* (Figure 1A), the equilibrium radius in the presence of spontaneous deviatoric curvature is given by $\text{r}=\sqrt{\kappa /\left(2\left(\lambda +\kappa {\text{D}}_{\text{m}}^{2}\right)\right)}$ (Supplementary Equation 37). Since this radius depends on both the value of tension and the spontaneous deviatoric curvature (Figure 5A), we define an effective tension ($\lambda +\kappa {\text{D}}_{\text{m}}^{2}$). As a result, the relationship between neck radius, spontaneous deviatoric curvature, and tension in Figure 5A collapses onto a single curve (Supplementary Figure 6B) as a function of this effective tension. Simulations confirm that the radii of tubular necks obtained from numerical solutions collapse onto a single curve as a function of effective tension (Figure 5B).

Similarly, the axial force required to maintain a tubular membrane with radius r and spontaneous deviatoric curvature D_m_ along the total length *L*, is given by ${\text{F}}_{\text{z}}=2\pi \left(\sqrt{2\kappa \left(\lambda +\kappa {\text{D}}_{\text{m}}^{2}\right)}-\kappa {\text{D}}_{\text{m}}\right)$ (Supplementary Equation 37). In Figure 5C, we plotted the axial force as a function of tension and spontaneous deviatoric curvature. We found that for a fixed value of tension, the axial force has a local minimum along the red line (Figure 5C) where λ =$\;\kappa {\text{D}}_{\text{m}}^{2}$ (Supplementary Equation 38) and F_z,min_ = 2πκD_m_ (Supplementary Equation 38). The 3D surface in Figure 5C can be reduced to a single curve by defining the effective axial force as F_z_+2πκD_m_ and plotting it as a function of effective tension (Supplementary Figure 6D). We also plotted the effective axial force obtained from numerical solutions as a function of effective tension (Figure 5D). We observed that consistent with the analytical prediction, for different tensions, the effective axial forces collapse onto a single curve as a function of effective tension (Figure 5D). These results suggest that effective tension ($\lambda +\kappa {\text{D}}_{\text{m}}^{2}$) regulates the radius of dendritic spine necks.

### 3.5. Cooperation of Forces and Induced Spontaneous Deviatoric Curvature Offers Multiple Pathways for Spine Shape Maintenance

Thus far, we have focused on the role of forces (axial and normal) on spine head shape and the role of spontaneous deviatoric curvature representing periodic rings on the spine neck radius. Next, we asked if the cooperation of these two different mechanisms could further influence the spine geometries and the energy landscape associated with these features. In other words, we asked if the combination of spontaneous deviatoric curvature and applied forces could result in lower energy states for the same spine geometry. To answer this question, we sought to identify the parameters that give rise to thin spines with the same geometric parameters. We explain this approach with a specific example below.

**Figure 6:**
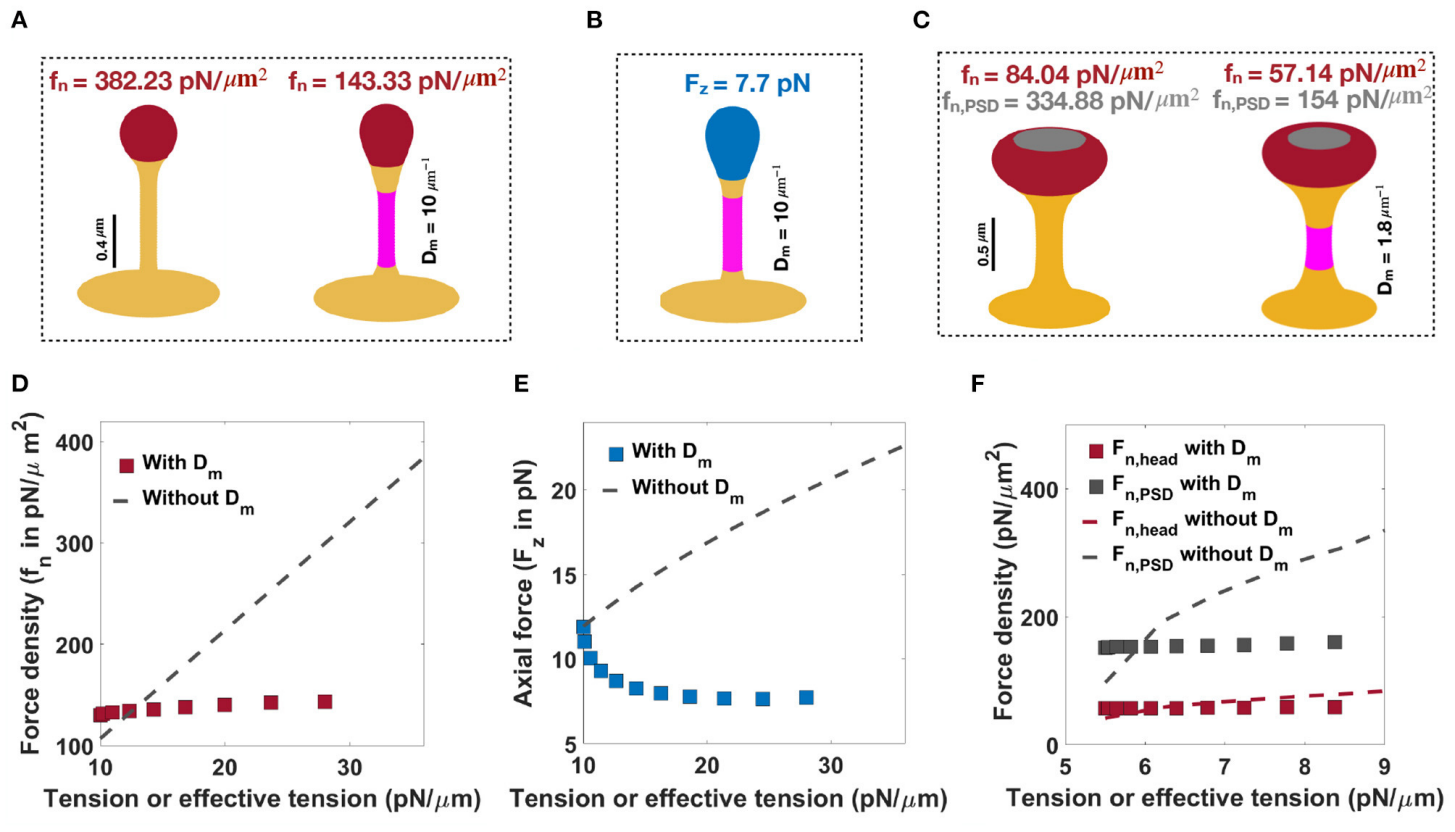
Formation of thin and mushroom shaped spines with a combination of forces and spontaneous deviatoric curvature. **(A)** Formation of a thin-shaped spine by applying a uniform normal force density along the spine head (left) vs. applying a uniform normal force density along the head and spontaneous deviatoric curvature (purple region) along the spine neck (right) (λ = 10 pN/μm). **(B)** Formation of a thin-shaped spine by applying an axial force along the spherical cap (blue region) and spontaneous deviatoric curvature along the spine neck (purple region), λ = 10 pN/μm. All thin spines in **(A,B)** have a neck radius *r* ~ 0.05 μm and head volume *V* ~ 0.033 μm^3^. **(C)** Formation of a mushroom-shaped spine by applying a non-uniform normal force density along the spine head (left) vs. applying a non-uniform normal force density along the head and spontaneous deviatoric curvature along the spine neck (purple region), (right), λ = 5.5 pN/μm. The formed mushroom spine with normal force densities f_n_ = 57.14 pN/μm^2^ and f_n,PSD_ = 154 pN/μm^2^ and deviatoric curvature D_m_ = 1.8 μm^−1^ has a neck radius r ~ 0.1 μm and head volume V ~ 0.27 μm^3^. **(D)** The magnitude of a normal force density that is required to form a thin-shaped spine with and without spontaneous deviatoric curvature as a function of effective tension and tension, respectively. **(E)** The magnitude of an axial force that is required to form a thin-shaped spine with and without spontaneous deviatoric curvature as a function of effective tension and tension, respectively. **(F)** The magnitude of normal force densities in the spine head and in PSD that is required to form a mushroom spine with and without spontaneous deviatoric curvature as a function of effective tension and tension, respectively.

As noted before, when only normal forces are used, a normal force density of f_n_ = 382.23 pN/μm^2^ under a tension of λ = 36 pN/μm is required to form a thin spine with a neck radius of *r* = 0.05 μm and head volume of *V* = 0.033 μm^3^ (Figure 6A, left). We can also obtain a thin spine with the same dimensions, by using a prescribed spontaneous deviatoric curvature D_m_ = 10 μm^−1^ along the neck and an applied force density of f_n_ = 143.33 pN/μm^2^ along the head for λ = 10 pN/μm (Figure 6A, right). Thus, for the same shape parameters, in the presence of spontaneous deviatoric curvature, the value of force density required is roughly one-third of the force density required in the absence of spontaneous deviatoric curvature (Figure 6A). Similarly, when a combination of axial force along the spine head and spontaneous deviatoric curvature along the neck is used, a thin spine with r ~ 0.05 μm and head volume V ~ 0.033 μm^3^ can be formed with F_z_ = 7.71 pN and spontaneous deviatoric curvature D_m_ = 10 μm^−1^ when λ = 10 pN/μm (Figure 6B). Thus, in both these cases (axial and normal forces) for the formation of thin spines, we note that access to spontaneous deviatoric curvature significantly reduces the forces required to form and maintain thin spines.

Not surprisingly, these same results hold for mushroom-shaped spines too. As we have shown before, to form a mushroom spine with a neck radius of *r* = 0.1 μm and head volume of *V* ~ 0.25 μm^3^, normal force densities of f_n_ = 84.04 pN/μm^2^ along the spine head and f_n,PSD_ = 334.88 pN/μm^2^ along the PSD are required under a tension of λ = 9 pN/μm (Figure 6C, left). We can also form a mushroom spine with the same dimensions and lower tension (λ = 5.5 pN/μm) by prescribing a spontaneous deviatoric curvature D_m_ = 1.8 μm^−1^ along the spine neck and normal force densities of f_n_ = 57.14 pN/μm^2^ and f_n,PSD_ = 154 pN/μm^2^ along the spine head and PSD, respectively (Figure 6C, right).

In Figures 6D–F, we plotted the magnitude of forces that are required to form thin and mushroom-shaped spines with or without spontaneous deviatoric curvature as a function of tension alone (with no spontaneous deviatoric curvature) or effective tension (with spontaneous deviatoric curvature). We observed that with increasing effective tension, the magnitude of the normal force density that is required to form a thin spine with spontaneous deviatoric curvature (red squares) is almost constant (Figure 6D). However, the magnitude of the normal force density that is needed to form a thin spine without spontaneous deviatoric curvature (dashed line) increases linearly with increasing tension (Equation (7) and Figure 6D). In the case of the formation of a thin spine with an axial force, we found that in the presence of spontaneous deviatoric curvature, the magnitude of axial force (blue squares) decreases slightly and then becomes constant with increasing effective tension (Figure 6E). In contrast, without spontaneous deviatoric curvature, the magnitude of axial force (dashed line) increases with increasing tension (Figure 6E). Similar to the thin-shaped spine, with spontaneous deviatoric curvature along the spine neck, the magnitude of normal force densities in the head (red square) and PSD (gray square) region that are required to form a mushroom spine is almost constant with increasing effective tension (Figure 6F). However, without spontaneous deviatoric curvature, the magnitude of force densities in both regions increases with increasing tension (Figure 6F).

**Figure 7:**
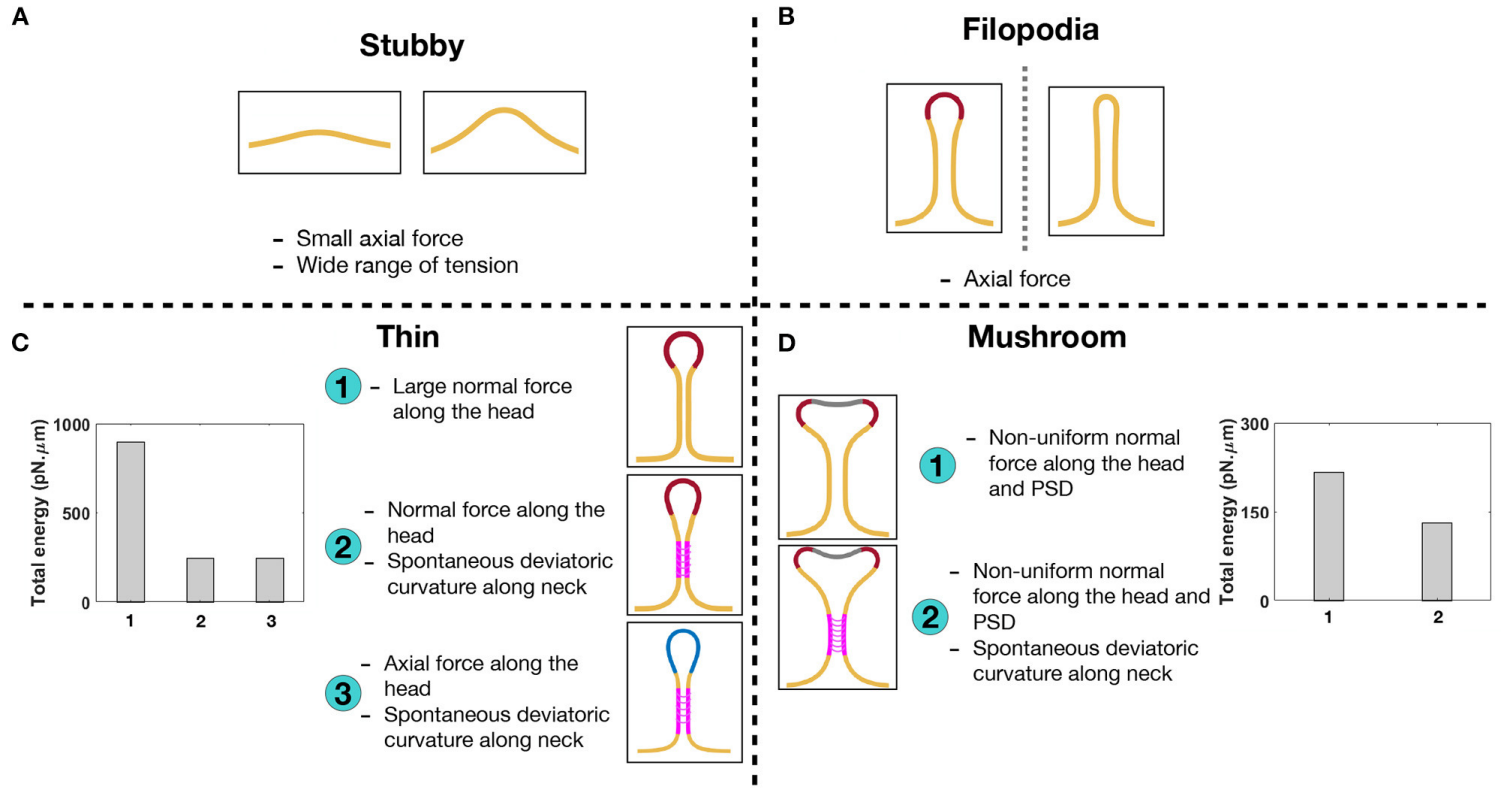
Characterizing different shapes of dendritic spines based on the mechanical model. **(A)** Stubby spines can be formed with an axial force and in a wide range of tensions. **(B)** An axial force is sufficient to form a long filopodial spine. **(C)** A thin-shaped spine can be formed with three different mechanisms; (1) a uniform normal force density along the spine head, (2) a uniform normal force density along the spine head and spontaneous deviatoric curvature along the neck, and (3) a uniform axial force density along the spine head and spontaneous deviatoric curvature along the neck. In the bar plot, the total energy of the system is shown for three different mechanisms. The total energy of the system for the second and third mechanisms with spontaneous deviatoric curvature is much less than the energy for the first mechanism with just a normal force. **(D)** A mushroom-shaped spine can be formed with two different mechanisms; (1) a non-uniform normal force density along the spine head and PSD region and (2) a non-uniform normal force density along the spine head and PSD region plus a spontaneous deviatoric curvature along the spine neck. The resulting mushroom spine with a combination of normal forces and spontaneous deviatoric curvature has lower energy compared to the spine that is formed with just normal forces (bar graph).

To further compare thin and mushroom spines shown in Figure 6, we computed the components of energy (Equation 1) and the total energy of the system for each shape (Supplementary Tables 2, 3 and Figures 7C,D). Based on our results, by prescribing spontaneous deviatoric curvature D_m_ along the spine neck, the bending energy due to deviatoric curvature decreases (Supplementary Tables 2, 3). This is because the deviatoric curvature *D* along the neck tends to D_m_ and minimizes the bending energy (Supplementary Tables 2, 3). Additionally, in the presence of spontaneous deviatoric curvature, in our simulation, we set the tension to lower values compared to the condition that D_m_ = 0. Therefore, the work that is done by tension and forces to bend the membrane reduces for the case that the spines obtained with a combination of force and spontaneous deviatoric curvature (Supplementary Tables 2, 3). For example, to form a thin spine shown in Figure 6, the work that is done by an axial force with a spontaneous deviatoric curvature (Figure 6B) is almost one third of the work that is done by a normal force without spontaneous deviatoric curvature (Figure 6A and Supplementary Tables 2, 3).

In the bar plots of Figures 7C,D, we compared the total energy of thin and mushroom spines formed with different mechanisms. We observed that in both thin and mushroom spines, the total energy of the system dramatically decreases when the spines form with a combination of forces and spontaneous deviatoric curvature (Figures 7C,D). This result suggests that spontaneous deviatoric curvature can alter the energy landscape of thin and mushroom dendritic spines to a lower energy state.

## 4. Discussion

Previous studies have showed that the coupled dynamics of signaling and actin remodeling can alter spine volume in a biophysical model (Rangamani et al., 2016) without considering the geometry of the spines or considering the role of spine shape in regulating different signaling pathways (Colgan and Yasuda, 2014; Yasuda, 2017; Bell et al., 2019; Rahmaninejad et al., 2020). In this work, we present a simplified mechanical model for studying the role of different force distributions and energy contributions that are associated with the different spine shapes noted in the literature. Our results show that different spine shapes can be associated with different forces and spontaneous deviatoric curvature distributions, giving us insight into the mechanical design principles of spine formation and maintenance (Figure 7).

We show that stubby spines can be formed for a wide range of tensions and low forces (Figure 2). From a spine formation viewpoint, this makes sense, since during development the stubby spines can be the initial protrusions that form out of the dendrites. Given the ubiquitous nature of stubby spines (Gray, 1959; Fiala et al., 1998; Harris, 1999), our results suggest that the prevalence of stubby spines could be due to the mechanical ease which they can be formed. They may also represent a temporarily stable state adopted by shrinking spines during synapse removal. Filopodia have the same force-length and force-radius relationships as membrane tubules that can be formed with micropipettes (Evans et al., 1996), optical tweezers (Raucher and Sheetz, 1999), or by kinesin motor proteins (Roux et al., 2002) (Figure 2).

Based on our results, dendritic filopodia can be formed with a relatively small axial force, which make them good candidates as initial protrusions for the formation of mature thin and mushroom spines. Thin and mushroom spines, which have defined head shapes, require more mechanical features—heterogeneous force distributions, normal or axial forces, and an induced spontaneous deviatoric curvature representing the periodic protein rings or other deviatoric curvature inducing mechanics along the neck. The heterogeneous distribution of actin-mediated forces and BAR domain proteins can be related to the nanoscale organization of actin filaments and protein phase separation on the membrane surface (Nowak et al., 2021; Yuan et al., 2021).

In the case of thin spines, we find that the mechanical design principles that support the formation of a spherical head are (1) large normal force along the head (Figure 3), (2) normal force along the head with a spontaneous deviatoric curvature along the neck (Figure 6A), and (3) an axial force along the head with a spontaneous deviatoric curvature along the neck (Figure 6B). Within these mechanisms, the presence of spontaneous deviatoric curvature significantly reduces the total energy of the spine (Figure 7C). Similarly, for mushroom spines, in addition to non-uniform forces along the head and the PSD (Figure 4), the spine can be formed with a combination of forces in the head and spontaneous deviatoric curvature along the neck (Figure 6C) while the spontaneous deviatoric curvature results in a lower energy state (Figure 7D).

These findings have implications for our understanding of how mechanical aspects of membrane dynamics such as bending, tension, membrane-protein interactions, and interactions of the membrane with the cytoskeleton play critical roles in spine geometry maintenance, particularly in structural plasticity. Many of the events associated with synaptic plasticity alter spine size and shape through changes in F-actin dynamics and the dynamics of the actin related proteins (Landis and Reese, 1983; Hotulainen and Hoogenraad, 2010; Bertling and Hotulainen, 2017). The net impact of changes in actin remodeling would likely result in changes in force distribution.

Another important and, as yet, under explored aspect of synaptic plasticity is the role of cortical membrane tension, including the effect of the membrane in-plane stresses and membrane-cytoskeleton interactions. We know that spines are sites of active vesicle trafficking events, such as endo- and exocytosis, and that these processes alter the membrane surface area and thereby alter the membrane tension (Blanpied et al., 2002; Collingridge et al., 2004). Here, we show that the effective membrane tension can play an important role in altering the energy required for the maintenance of different spine shapes. One of the main impacts of such effective tension is that because of the cooperative effects of spontaneous deviatoric curvature and the applied forces, the energy required to maintain certain spine shapes may be lower. Thus, we show that there are different mechanical pathways that are likely associated with the different spine shapes and that some mechanisms may be energetically more favorable than others.

Despite these insights, our model has certain limitations. We do not explicitly consider the remodeling of the actin network or the dynamics of the associated proteins, but use force as a lumped parameter. Additionally, the use of axisymmetric coordinates restricts our ability to obtain realistic spine shapes (Lee et al., 2020).

The impact of mechanical aspects of actin remodeling and membrane mechanics on structural plasticity is highly intriguing and we are only beginning to understand their effects on spine functionality. This complexity is immediately apparent in dendritic spines, which undergo dynamic changes, both mechanical and biochemical during structural plasticity spatiotemporal scales. For example, it is known that electromagnetic forces can alter membrane shape either through signaling or direct forces (Tasaki and Byrne, 1992; El Hady and Machta, 2015). The change in the geometry of the spine affects the membrane capacitance and ultimately neural activities (Ma et al., 2019). While in our model we do not consider the induced forces from time-dependent neural activities such as a change in membrane potential, the electrostatic contributions have been implicitly considered in the membrane elastic properties and induced spontaneous curvatures (Winterhalter and Helfrich, 1988; Andelman, 1995; Steigmann and Agrawal, 2016; Tarun et al., 2020). We believe that our minimal model provides insights into the possible mechanical aspects underlying the characteristic geometries associated with dendritic spines. This is an important step toward deciphering the intricate mechanochemistry of structural plasticity and dendritic spine development.

## Data Availability Statement

The raw data supporting the conclusion of this article will be made available by the authors, without undue reservation.

## Author Contributions

HA and PR conceived the research. HA, MB, and PR conducted the research and analyzed the data. HA, MB, SH, and PR wrote the paper. All authors reviewed the manuscript and agreed on the contents of the paper.

## Conflict of Interest

The authors declare that the research was conducted in the absence of any commercial or financial relationships that could be construed as a potential conflict of interest.
